# Supplementary material for: A Simple 3D Cell Culture Method for Studying the Interactions between Human Mesenchymal Stromal/Stem Cells and Patients Derived Glioblastoma
Source: Cancers (Basel). 2023 Feb 18;15(4):1304. doi: 10.3390/cancers15041304 (PMC9954562; doi:10.3390/cancers15041304)
Supplement: Supplementary file 1 [file cancers-15-01304-s001.zip › cancers-2199259-supplementary.pdf]

GBM	Type	Morphology	Subtype	Treatment	Survival
GBMA1	GBM IV	neurospheres	mesenchymal	Stupp	unknown
GBM3	GBM IV	adherent	proneural	Stupp	13 m
GBM8	GBM IV	neurospheres	classical	Stupp	18 m
GBM22	GBM IV	neurospheres	mesenchymal	Stupp	15 m
GBM69	GBM IV	neurospheres	classical	Stupp	17 m

Table S1: list of patients derived GBM used in this study with morphological and subtypes.
For all patients, surgery was followed by combined chem and radiotherapies (Stupp et al.Lancet Oncol. 2009;10(5):459-66. doi: 10.1016/S1470-2045(09)70025-7).
Patients survival was within the normal range observed with GBM.

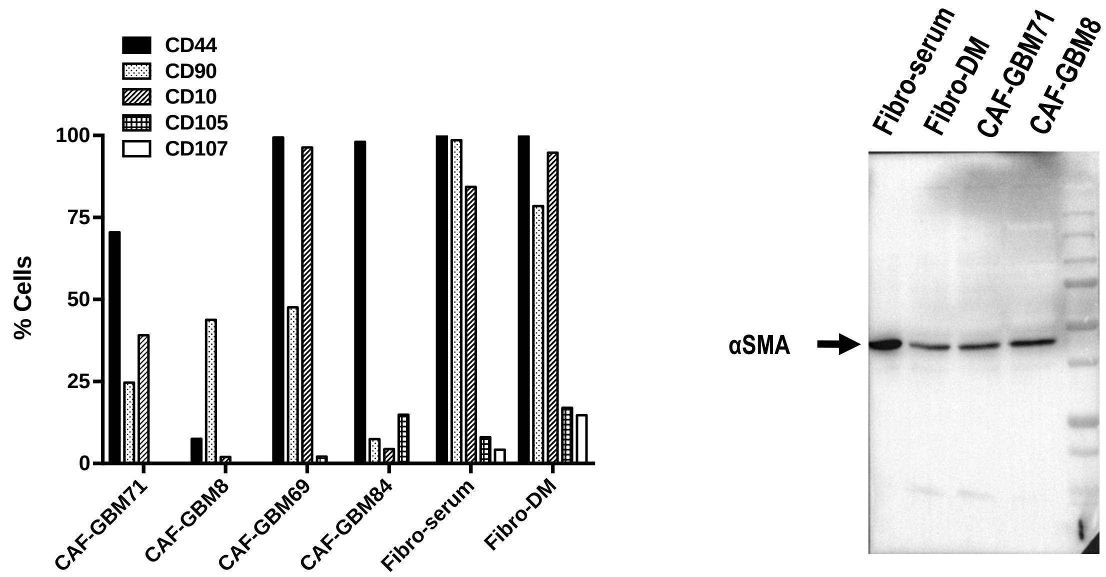

Figure supl. S1:

FACS analyses of the different CAF populations prepared with different primary culture conditioned media versus fibroblasts using antibodies directed against CD44, CD90, CD10, CD105 and CD107 (markers used to characterized fibroblasts) and showed marked differences in the expression of these markers between the different CAFs tested (left) while all the different CAF populations expressed αSMA (right)

GBMXX: patients derived primary cultures.

Fibro-serum: fibroblasts cultured in fetal calf serum.

Fibro-DM: fibroblasts cultured in defined medium.

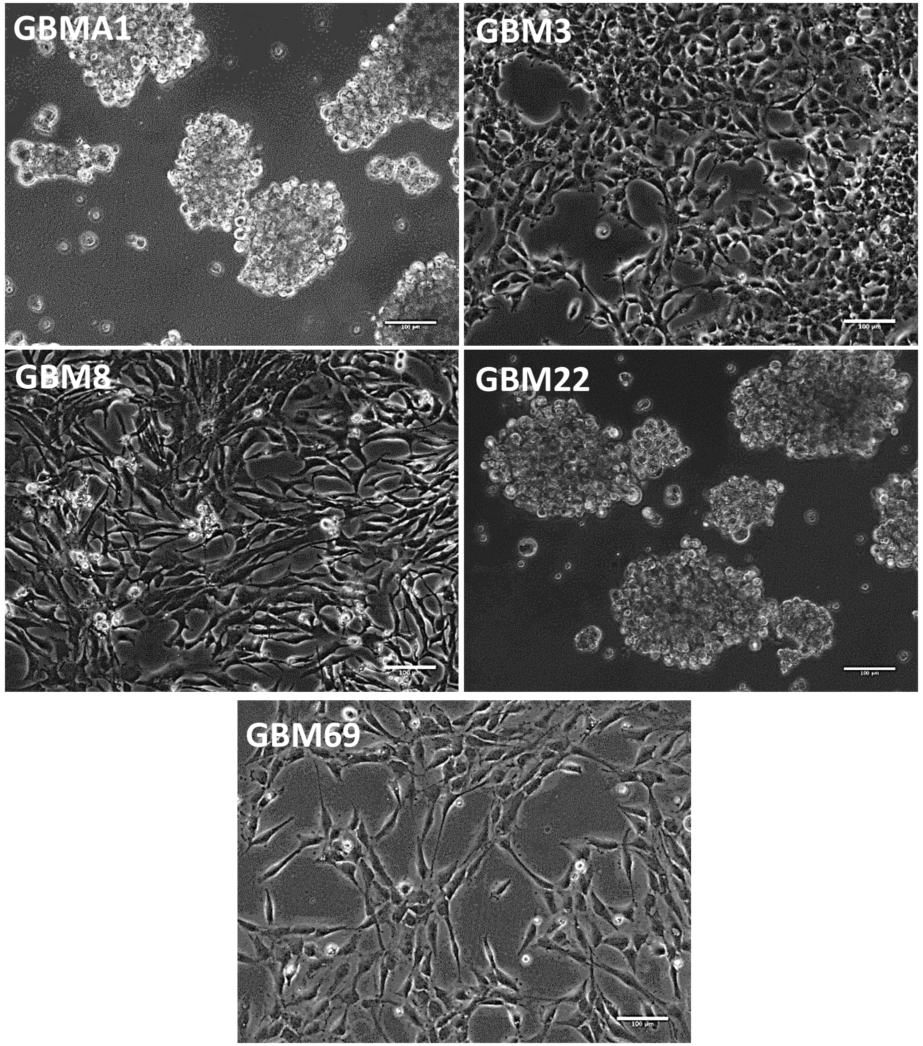

Figure Suppl S2:
Cellular netwoks of GBM grown under 2D conditions which are more divers and when spheroids are formed less compact
